# Use of wound infusion catheters for postoperative local anaesthetic administration in cats

**DOI:** 10.1177/1098612X231193534

**Published:** 2023-09-15

**Authors:** Kristina Kazmir-Lysak, Lucía Torres-Cantó, Sara Ingraffia, Giorgio Romanelli, Federico Massari, Matteo Rossanese, Krizia Compagnone, Guido Pisani, Filippo Cinti, Vincenzo Montinaro, Francesco Collivignarelli, Sayaka Okushima, Rosario Vallefuoco

**Affiliations:** 1 Section of Anaesthesiology, Department of Clinical Diagnostics and Services, Vetsuisse Faculty, University of Zurich, Zurich, Switzerland; 2 Anaesthesia Department, Southern Counties Veterinary Specialists, Ringwood, Hampshire, UK; 3 Centro Specialistico Veterinario, Milano, Italy; 4 Clinica Veterinaria Nervianese, Nerviano, Italy; 5 Royal Veterinary College, Hatfield, Hertfordshire, UK; 6 Davies Veterinary Specialist, Higham Gobion, Hertfordshire, UK; 7 Centro Veterinario Luni Mare, Luni, Italy; 8 Clinica San Marco, Veggiano, Italy; 9 Clinica Veterinaria Malpensa, Samarate, Italy; 10 Faculty of Veterinary Medicine, University of Teramo, Teramo, Abruzzo, Italy; 11 Pride Veterinary Referrals, Derby, UK

**Keywords:** Local anaesthetics, wound infusion catheter, complications, multimodal analgesia

## Abstract

**Objectives:**

The present study aimed to document the use of the wound infusion catheter (WIC) following a variety of surgical procedures in cats, investigating complications and risk factors associated with catheter placement or local anaesthetic (LA) administration.

**Methods:**

A retrospective, multicentric study was performed. Medical databases of eight veterinary referral hospitals from 2010 to 2021 were searched to identify records of cats where WICs were used. Information regarding signalment, type of surgery, size and type of WIC placed, and LA protocol used, as well as postoperative complications, were retrieved.

**Results:**

One hundred and sixty-six cases fulfilled the inclusion criteria. Feline injection site sarcoma resection was the most common surgery. Overall complications were identified in 22/166 cats (13.2%). Thirteen cats (7.8%) experienced wound-related complications, whereas nine cats (5.4%) experienced drug-delivery complications. The only factor associated with an increased risk of complications was the amount of a single dose of LA delivered through the catheter (*P* <0.001). An amount higher than 2.5 ml of LA delivered at each administration was associated with an increased risk of complications. All complications were minor and self-limiting.

**Conclusions and relevance:**

WICs were used for a large variety of surgical procedures with different protocols of LA administration as part of a multimodal analgesic plan in cats. The risk of complications was relatively low and self-limiting, suggesting its safe use in cats. Further prospective studies are required to evaluate efficacy of postoperative analgesia and to determine the suitable protocol for WIC handling and maintenance.

## Introduction

Pain is a distressful sensation after a stimulus and is usually unique for each individual.^1^ The failure to detect and manage it adequately may lead to increased postoperative stress and arterial blood pressure, immunosuppression, delayed wound healing, negative protein balance, decreased food intake and development of maladaptive behaviours, including self-mutilation.^2,3^

Opioid analgesics have been the mainstay of pain management, but other medications, such as alpha_2_-adrenoceptor agonists, dissociative anaesthetics, tranquilisers, non-steroidal anti-inflammatory drugs and local anaesthetics (LAs), have been used to complement opioid treatment.^4,5^ A multimodal approach incorporating LA administration is becoming more popular as means to provide analgesia.^6–8^ Local anaesthetics are unique because they can completely block the transmission of nociceptive signals, resulting in local anaesthesia and analgesia.^9^ They work by blocking the sodium influx to the nerve axon and inhibiting the action potential, which disrupts the generation and transmission of nerve impulses.^10^ In addition, unlike other drugs such as opioids that modulate pain through receptor-binding in the central nervous system (CNS), LAs prevent nociceptive impulses from reaching the CNS, enabling specific and powerful control of the nociceptive pathway.^11^

The administration of LAs through a soaker catheter maximises their effects by allowing repeated or continuous administration, and facilitates blocking somatic sensation of the incision and surrounding muscle beds after surgical procedures.^6^ A soaker catheter, also known as a wound infusion catheter (WIC), has a relatively low cost and results in minimal LA systemic absorption.^12,13^ Placement of a WIC directly into the surgical wound is technically easy. To date, these systems have been effective in providing postoperative analgesia to human patients undergoing a wide variety of orthopaedic and soft-tissue procedures.^14–16^ Numerous benefits including shorter hospitalisation, decreased cost, fewer systemic side effects and a decreased need for supplemental systemic analgesic drugs have been recognised in human patients.^17–20^ In the past decade, this method has been adopted as a postoperative analgesia in dogs, cats and goats.^6,13,21–23^ However, to our knowledge, only a few studies on the use of WICs in feline patients have been published with a limited number of cases.^6,21^ Furthermore, protocols and complications have not always been fully detailed.^6,21^

The objective of this retrospective study was to evaluate the use of the WIC following a variety of surgical procedures in cats investigating complications and risk factors associated with WIC placement and LA administration.

## Materials and methods

### Study design and eligibility criteria

Medical records from eight veterinary referral hospitals were retrospectively searched to identify client-owned cats in which WICs were used as part of multimodal postoperative analgesic regimen between January 2010 and December 2021. Cases were excluded from the study if the records were incomplete. The data retrieved from the medical records comprised: breed, age, sex, weight, type of surgery, size and type of WIC placed, application of a filter, WIC location (subcutaneous vs intramuscular), time (h) from WIC placement to removal, type of LA, type of LA administration (continuous vs intermittent), total dose (mg) and total amount (ml) of LA delivered (<2.5 ml vs >2.5 ml), frequency and single dose amount (ml) of LA delivered and any complications encountered until the recheck. The rechecks were performed by a veterinarian at the referral institution or at a referring practice at 13–15 days from the surgery or later in case of complication. Complications were classified as wound-related and drug delivery complications.^23^ Wound-related complications included fluid accumulation/drainage, localised swelling at the suture site, diffuse oedema of the surgical site, seroma, wound dehiscence and surgical site infection. Postoperative surgical site infection was defined according to Centers for Disease Control and Prevention as a post-surgical infection that occurs within 30 days of the surgical procedure (or within 1 year of an implant placement) and must include at least one of the following features: purulent debris; positive bacterial culture; or pain, swelling, fever and redness at the surgical site.^24,25^

Drug delivery complications included local (local skin reaction or pain at injection) or systemic toxicity (emergence delirium, dysphoria, neurological signs, uncontrolled pain, respiratory distress) and any technical problems associated with the WIC (catheter dislodgement, occlusion, loss of negative pressure or resistance during injection). Emergence delirium was defined as a state of mental confusion and psychomotor agitation marked by hyperexcitability, restlessness, uncontrolled thrashing and vocalisation.^26^

### Statistical analysis

Analyses were performed using Excel 2021 (Microsoft) and SPSS, version 26.0 (IBM). Descriptive statistics were reported for all variables. The Shapiro–Wilk test confirmed that the data were not normally distributed (*P* <0.001) and all the data were reported as median and range. Assessed continuous explanatory variables were age, body weight, WIC size, single dose amount (ml) and frequency of LA administration, total LA dose (mg), total LA amount delivered (ml) and time (h) from WIC placement to removal. Assessed categorical variables were sex, neuter status, type of surgery, application of a WIC filter, WIC location (subcutaneous vs intramuscular), type of LA (bupivacaine vs ropivacaine), type of LA administration (continuous vs intermittent), use of NSAIDs and occurrence of postoperative complications.

Simple and multiple logistic regression were used to determine the association of a range of variables with the occurrence of complications. The outcome variables were the occurrence of any type of complication associated with the WIC, and the explanatory variables were those listed above. These variables were first tested separately with simple logistic regression. A multiple logistic model was then built, which initially included the variables identified as *P* <0.2 by simple regression. The model was then refined over multiple rounds, using backward-stepwise elimination of the least significant variable each time, and variables were only retained in the final model if they were significant in their own right (*P* <0.05). Logistic regression results were reported as odds ratios, 95% confidence interval and the associated *P* value. *P* <0.05 (two-sided) was considered statistically significant.

## Results

In total, 210 medical records of cats in which WICs were used were retrospectively evaluated. Only 166 cats met the eligibility criteria for the study after the exclusion of 44 cats, owing to the incomplete or inadequate medical records and follow-up. The most represented breeds were domestic shorthair (n = 138), followed by British Shorthair (n = 6), Maine Coon (n = 4), Bengal (n = 4), domestic longhair (n = 2) Persian (n = 3), Norwegian Forest Cat (n = 2), Siamese (n = 2), Turkish Van (n = 2), Charteuse (n = 1), Egyptian Mau (n = 1) and Soriano (n = 1). The median (range) age was 10 (0.5–17) years and their median (range) weight was 5 (1.4–10.2) kg. There were 81 intact and three castrated males together with 80 intact and two spayed females. The most common surgical procedure was feline injection site sarcoma excision (58.4%, n = 97), followed by limb amputation (16.8%, n = 28), thoracotomy (12%, n = 20), mastectomy (7.22%, n = 12), abdominal wall resection (2.4%, n = 4), other neoplasia removal (2.4%, n = 4) and joint stabilisation (0.6%, n = 1). The median (range) size of the catheter used was 4 (2–9) Fr. Mila WIC (MILA International) was used in 159 cats (95.7%), Dahlhausen WIC (Dahlhausen & Co.) was used in five cats (3%), a modified red rubber catheter (Tyco) was used in one cat (0.6%) and a modified rhinogastric catheter (Tyco) was used in one cat (0.6%). The filter (MILA International) was employed in 151 cats (90.9%).

The WIC was placed within the muscular layers in 85 cases (51.2%) and in the subcutaneous tissue in 81 cases (48.8%). The WIC was left in place for a median (range) of 45 (2.5–120) h. Bupivacaine 0.25% and 0.5% were used in six (3.6%) and 105 (63.2%) cats, respectively. Ropivacaine 0.25%, 0.5% and 0.75% were used in two (1.2%), 29 (17.4%) and 24 (14.45%) cats, respectively. In all cases, the LA was administrated intermittently with a median (range) frequency of 6 (1–12) h. The administered dose of bupivacaine and ropivacaine was 1.06 (0.48–17.00) mg/kg and 1.49 (range 0.73–2.00) mg/kg, respectively. The total volume of LA was 7.7 (0.82–195.00) ml and 6.36 (0.84–17.92) ml for bupivacaine and ropivacaine, respectively. The volume of a single dose of LA delivered per each administration was 1.3 (0.34–15) ml for bupivacaine and 1 (0.28–2.26) ml for ropivacaine.

Complications were identified in 22 cats (13.2%). Thirteen cats (7.8%) experienced wound-related complications: diffuse surgical site oedema (4.2%, n = 7), seroma (1.8%, n = 3), localised swelling at level of the suture site (0.6%, n = 1) and surgical wound dehiscence (1.2%, n = 2). Wound-related complications occurred in 11 (6.6%) cats that had undergone feline injection site sarcoma excision (Table 1).

**Table 1:** Complications identified in 166 cats undergoing different types of surgery

Surgery type	Number of animals	Wound-related complication	Drug delivery complication
FISS excision	97	2 seroma 7 oedema 2 dehiscence	2 dislodgement 1 resistance to injection
Mastectomy	12	1 swelling	1 dislodgement 1 resistance to injection
Limb amputation	28	0	1 local irritation during injection 1 hypersalivation
Thoracotomy	20	1 seroma	1 dislodgement 1 resistance to injection
Abdominal wall resection	4	0	0
Other neoplasia excision	4	0	0
Joint stabilisation	1	0	0
Total	166	13	9

FISS = feline injection site sarcoma

Nine cats (5.4%) experienced drug-delivery complications: local pain at injection of LA (0.6%, n = 1), hypersalivation after administration of LA (0.6%, n = 1) and technical issues (4.2%, n = 7). Technical issues included: catheter dislodgement (2.4%, n = 4) and resistance during injection (1.8%, n = 3) (Table 1).

Logistic regression analysis was used to determine factors associated with the occurrence of complications, when considering possible confounding factors (Table 2). After the initial model was refined by backward-stepwise elimination, the best-fit model was one that included three variables. In the final multiple regression model (Table 3), the only factors positively associated with an increased risk of complications was the amount of local anaesthetic delivered through the catheter (*P* <0.001). An amount higher than 2.5 ml of single dose of LA delivered at each administration has been found to be associated with an increased risk of complications.

**Table 2:** Simple logistic regression results determining factors associated with all complications after surgical wound infusion catheter placement in cats

Logistic regression	All complications		
	OR	95% CI	*P* value
Age	0.99	0.98–1.00	0.339
Gender	**2.73**	**0.70–10.70**	**0.148**
Body weight	1.06	0.69–1.61	0.791
Type of surgery	1.20	0.69–2.09	0.515
Catheter size	0.94	0.53–1.66	0.843
Use of a filter	0.44	0.80–2.27	0.332
Catheter location (subcutaneous vs intermuscular)	1.15	0.33–3.94	0.820
Catheter duration	0.99	0.97–1.00	0.352
Type of LA (ropivacaine vs bupivacaine)	0.40	0.84–1.93	0.257
LA concentration	**0.43**	**0.12–1.52**	**0.191**
LA administration frequency	1.20	0.80–1.80	0.365
LA total dose	1.00	0.99–1.00	0.249
LA single dose	**5.44**	**1.55–19.10**	**0.008**
Use of NSAID	3.78	0.47–30.47	0.211

Variables highlighted in bold qualified for inclusion in the multiple regression analysis at *P* <0.20 (Table 3). OR = odds ratio; CI = confidence interval: reference category used in logistic regression; LA = local anaesthetic; NSAID = non-steroidal anti-inflammatory

**Table 3:** Multiple logistic regression results determining factors associated with all complications after surgical wound infusion catheter placement in cats

Logistic regression	All complications		
	OR	95% CI	*P* value
Gender	2.59	0.64–10.42	0.180
LA concentration	0.53	0.13–2.16	0.380
LA single dose	5.35	1.52–18.80	0.009

OR = odds ratio; CI = confidence interval; LA, local anaesthetic

## Discussion

This is the first retrospective, multicentric study with the aim of documenting the use of the WIC following a variety of surgical procedures in cats, investigating any complications and risk factors associated with catheter placement or LA administration.

The results of the study document the versatile and safe use of the WIC for a large variety of surgical procedures, for which LA administration was used as part of a multimodal analgesic plan in cats. The WIC was used with different protocols of LA administration, and it was left in place for variable durations.

The reported overall complications rate in the present study was relatively low (13.2%) and all complications were self-limiting. Wound-related complications occurred in 7.8% of cases, the majority of which were observed with the feline injection site sarcoma excision. However, statistical analysis failed to demonstrate significant association between the complications and type of surgery. This result could have been influenced by the high number of cases of feline injection site sarcoma removal compared with the other type of surgery (type II error). Feline injection site sarcomas are locally invasive tumours and require aggressive surgical treatment. Radical surgical excision is challenging, with the current recommendations being 5 cm lateral margins and two fascial planes for deep margins.^27^ This can be associated with a higher incidence of postoperative wound-related complications.

In the present study, drugs delivery complications were encountered in 5% of cases. Technical issues (catheter dislodgement and resistance during injection) were reported in seven cases. The external location of the WIC makes it more exposed to mechanical interference during the hospitalisation time. In both human^28^ and veterinary studies,^6,23^ it has been reported that the catheter can be dislodged, disconnected or partially blocked at the outlet.

The results of the present study suggest that the only risk factor associated with the overall complications was the amount of a single dose of LA delivered through the catheter. A volume higher than 2.5 ml of LA delivered at each administration has been found to be associated with an increased risk of complications. Such a finding should be interpreted cautiously because it does not factor the wound size and the speed of administration relative to the amount of LA administrated. However, it would be logical to assume that a larger volume of LA drugs would require more time to be absorbed and could cause seroma, oedema or wound swelling. This finding is in contrast to the previously published data in human patients^29,30^ and veterinary medicine^23^ where the incidence of wound-related complications did not relate to the volume, rate or drug content of the LA infusion.

One cat experienced local irritation and another experienced hypersalivation at time of the administration of LA. In both cases, the LA used was bupivacaine 0.5%. Tissue reactions induced by the LA solutions may be one of the factors resulting in pain after application.^31^ Based on a study conducted on human volunteers, it has been determined that the pain experienced during intramuscular injection of bupivacaine 0.5% is significantly more intense compared with ropivacaine 0.5%.^32^ Interestingly, the variance in pain intensity between these two LAs does not appear to be associated with differences in pH.^32^ Lipid solubility of the LA has also been considered as a factor in the severity of pain on injection.^33^ This factor provides justification for the observation that bupivacaine, being more liposoluble compared with ropivacaine, may cause greater pain during injection. However, there is insufficient evidence to support this statement in veterinary literature. Although the cause of pain after LA injection is not fully understood, adding a basic solution (typically sodium bicarbonate) to the LA solution before injecting it into the target tissues may decrease the pain on injection.^34^

The hypersalivation that presented in one cat could be compatible with signs of neurotoxicity; however, we cannot completely rule out other causes. Cats are more sensitive to LA systemic toxicity, which can be explained by their reduced hepatic metabolism;^35^ therefore, there is an existing concern during prolonged administration. Recently, local anaesthetic systemic toxicity associated with bupivacaine administration has been reported in two cats.^36,37^ In the first case, bupivacaine was accidentally overdosed (10 mg/kg) during intrapleural administration.^36^ In the second case, bupivacaine was delivered epidurally through an epidural catheter over several days, causing toxicity due to accumulation.^37^ However, there are no reports of severe neurotoxicity after subcutaneous or intramuscular administration of LA in cats.

The LA drugs used in this retrospective study were bupivacaine and ropivacaine at different concentrations. Bupivacaine and ropivacaine are aminoamide LAs with a slow onset and a long duration of action. Bupivacaine compared to the *S*-enantiomer ropivacaine, is more lipophilic and potent than ropivacaine and, consequently, it is more neurotoxic and cardiotoxic.^38^ In cats, the mean convulsant dose was 3.8 ± 1.0 mg/kg IV and 18.4 ± 4.9 mg/kg IV for cardiovascular collapse.^39^ In the present study, the administered dose closely adhered to those described in the literature. The recommended doses are 1 mg/kg for bupivacaine, whereas the dose for ropivacaine is 1–2 mg/kg in cats.^40^

In humans, the reported incidence of LA toxicity after different nerve blocks varied across studies, with estimates ranging from as low as 2.5 cases per 10,000 blockades to as high as 10 cases per 10,000 blockades.^41–43^ Notably, one study recorded no events in over 12,000 blockades.^44^ The incidence of systemic toxicity in veterinary species is not documented, but is probably very low.^11^

Recommendations for pain management encourage the use of LAs in the majority of surgical procedures.^7,8^ The combination of LA and systemic opioids not only improves pain management, but also allows a decrease in the opioid dosage, thereby decreasing the risk of adverse effects associated with opioid administration, such as bradycardia, respiratory depression, hypothermia and sedation.^5,45,46^ LAs have been administered perineurally, epidurally, intrapleurally, intra-articularly and topically to alleviate pain associated with various surgeries in dogs.^46–48^ There is growing evidence in the human and porcine models that locally applied LAs can also inhibit the inflammatory responses that can sensitise nociceptive receptors and contribute to the development of pain and hyperalgesia.^49,50^

The use of WICs offers an additional benefit by allowing repeated LA administration throughout the postoperative period. Although postoperative pain score and food intake evaluation were beyond the scope in the present study, a previous study reported that cats receiving LAs infused through wound catheters spent significantly less time in hospital than those that did not, suggesting that the cats became mobile more quickly and took less time to start eating than those on other analgesic regimens.^21^ Similar results have been observed in human studies, where the use of WICs with LAs led to a reduced hospital stay of 2.1 days compared with 3.2 days in control patients who received systemic analgesia alone, resulting in significant cost savings.^51^ However, further research is needed in both human and veterinary fields to validate this finding.

The main limitation of the present study is the retrospective and multicentric nature with a lack of standardised postoperative reporting. Multiple protocols and different LAs (bupivacaine and ropivacaine) were used at different concentrations. Moreover, the study population included patients undergoing a wide range of procedures, introducing additional confounders related to underlying surgical pathology and technique.

Further prospective studies are required to evaluate the efficacy of postoperative analgesia, to determine the optimal amount and concentration of LA drug administrated and to describe the suitable protocol for WIC handling and maintenance aiming to optimise the analgesia at the same time as avoiding complications.

## Conclusions

Based on the findings of the present study, use of the WIC can be considered as part of the multimodal analgesic approach for postoperative pain management in cats. The placement of the WIC can be easily performed by the attending surgeon at the end of surgery in a large variety of surgical procedures with different LA administration protocols. The low incidence of major complications in this population of cats illustrates that the use of WICs is safe and encouraging.
